# Editorial: Advances in imaging and treatment of embolic stroke of undetermined source

**DOI:** 10.3389/fneur.2022.1073545

**Published:** 2022-11-30

**Authors:** Bing Tian, Xia Tian, Chengcheng Zhu

**Affiliations:** ^1^Department of Radiology, Changhai Hospital of Shanghai, Shanghai, China; ^2^Department of Radiology, University of Washington, Seattle, WA, United States

**Keywords:** embolic stroke of undetermined source, embolic stroke, intracranial atherosclerotic plaque, imaging, antithrombotic, antiplatelets

Determining the causes of acute ischemic stroke is crucial for patient management, particularly to prevent future strokes. Embolic stroke of undetermined source (ESUS) is a subtype of cryptogenic ischemic stroke and accounts for ~20% of all ischemic stroke, and the average recurrence rate of 4.5% per year is relatively high, despite preventive treatment with antithrombotic agents (1). Recent studies have shown that novel clinical strategies and imaging techniques can improve the detection rate of the etiology of ESUS.

## Atherosclerosis and ESUS

Non-stenotic atherosclerotic plaques are a major contributor to ESUS (2). Several previous studies revealed that certain morphological features of plaques on ultrasound, computed tomography (CTA), or high-resolution MRI vessel wall imaging (HR-VWI) are significantly more common in ESUS patients ipsilateral to the side of stroke than the contralateral side (3). High-risk culprit plaque can still present despite the stenosis degree being <50%, and the outward remodeling of the plaque is common. HR-VWI provides the opportunity to image the high-risk features of the plaques with <50% stenosis, including intraplaque hemorrhage, lipid-rich necrotic core, and thin or ruptured fibrous caps (4).

In this Research Topic, Che et al. concluded that extracranial carotid intra-plaque hemorrhage (IPH) on HR-VWI was significantly associated with poor 3-month outcomes after acute ischemic stroke and could predict a poor 3-month functional prognosis. Baradaran et al. discussed the role of cross-sectional imaging of the extracranial and intracranial arteries and how imaging might potentially uncover high-risk plaque features that may be contributing to ischemic strokes. They highlighted that, in the extracranial carotid artery, both MR and CTA could be used to identify certain plaque features which indicated more plaque vulnerability including IPH on MR and increased soft plaque thickness on CTA. VW-MR can also be used as a powerful tool to identify active atherosclerotic plaque in the intracranial arteries with <50% stenosis by identifying an enhancing plaque with positive remodeling. Lin et al. found the culprit plaque characteristics of patients with symptomatic MCA atherosclerotic in different stroke mechanisms may be evaluated using HR-VWI. The plaque characteristics of different stroke mechanisms may have clinical value for the selection of treatment strategies to prevent stroke recurrence.

There has been increased attention given to decoding the contributory role of non-stenotic atherosclerosis in the carotid arteries, intracranial arteries, and the aortic arch in ESUS patients. Cai X. et al. found that aortic arch calcification, especially spotty calcification, had a good predictive value for stroke recurrence in patients with ESUS. Sakai et al. reviewed the use of VW-MRI in detecting and characterizing carotid, intracranial, and aortic arch atherosclerosis in ESUS patients. Besides high-risk feathers of carotid and intracranial atherosclerosis, HR-VWI also allows further characterization of aortic arch plaque compositions like that seen in carotid studies.

## Cardioembolic and ESUS

Occult atrial fibrillation (AF) is a leading cause of stroke of unclear cause (5). Chen C.-H. et al. revealed that MRI at stroke onset provides critical clues for the prediction of newly detected AF, including single cortical infarcts, territorial infarcts, and early hemorrhage. Future studies are warranted to verify their new prediction model and to assess whether the identification of AF can be enhanced to improve outcomes after acute ischemic stroke. Hou et al. found that potential embolic sources (PES) differ in patients with ESUS according to age and differences in recurrence. PFO is the only common PES in young patients with ESUS. Future studies prospectively evaluating PES in both age groups are needed. Chen L. et al. provided a case of cryptogenic stroke associated with infective endocarditis (IE) and antiphospholipid antibody syndrome (APS). They pointed out that bicuspid aortic valve (BAV) vegetation-related cerebral embolism might present as cryptogenic and can be confusing in the acute phase, particularly when APS and IE were diagnosed simultaneously. Kato and Takahashi provided a review of atrial cardiopathy and cryptogenic stroke. They concluded that atrial cardiopathy should be considered as one of the mechanisms of ESUS. Abnormal atrial substrate (atrial cardiopathy) that leads to atrial fibrillation (AF) can result in embolic stroke before developing AF and may explain the source of cryptogenic stroke in some patients.

## Other research in ESUS

There are many other biomarkers associated with ESUS. Cai Z. et al. outlined the current understanding of the regulatory network of non-coding RNAs (ncRNAs) and reviewed the recent evidence for the contribution of ncRNAs in the experimental ischemic stroke model. Zhou et al. pointed out that carotid web (CaW) was a risk factor in cryptogenic stroke because it could be detected in nearly 5% of young cryptogenic stroke patients. Contrast-enhanced ultrasound (CEUS) might have higher diagnostic accuracy for CaW with thrombosis, and it had a higher clinical application prospect. Dong and Ma reviewed advances in exploring uncommon female-predominant etiologies of cryptogenic stroke. This review provided novel clinical clues for the etiological diagnosis of cryptogenic stroke and will help to improve the management and secondary prevention of stroke in the female population.

## Future directions

Though these studies can be helpful in determining the source of potential emboli for ESUS, further studies are needed to validate these imaging techniques and pave a path for their routine use in clinical practice. The use of VW-MRI to detect and characterize carotid, intracranial, and aortic arch atherosclerosis in ESUS patients is an exciting and rapidly evolving field. Additional efforts are warranted to elucidate the contributory role of these atherosclerotic plaques in ESUS. The ARCADIA trial is currently being performed to validate the diagnosis of atrial cardiopathy and to determine whether atrial cardiopathy can be a new therapeutic target for direct-acting oral anticoagulants (DOAC). It was also hypothesized that oral anticoagulation may decrease the risk of stroke recurrence in ESUS, which was tested in two large randomized controlled trials: the NAVIGATE ESUS and the RE-SPECT ESUS (6, 7). For ESUS patients without atherosclerotic lesions, it seems rational to hypothesize that oral anticoagulation could reduce the risk of stroke recurrence. But for ESUS patients with atherosclerosis, further research is needed to prove the combined use of low-dose anticoagulation with antiplatelets.

## Author contributions

BT: writing—original draft and funding acquisition. XT: writing—original draft. CZ: writing—review and editing and funding acquisition.

## Funding

This research activity is funded by the Medical Guidance Project of the Shanghai Science and Technology Commission (1941196500) and the Nature Science Foundation of Shanghai (21ZR1479300). CZ was supported by the US National Institute of Health (NIH) Grants R01HL162743 and R00HL136883.

## Conflict of interest

The authors declare that the research was conducted in the absence of any commercial or financial relationships that could be construed as a potential conflict of interest.

## Publisher's note

All claims expressed in this article are solely those of the authors and do not necessarily represent those of their affiliated organizations, or those of the publisher, the editors and the reviewers. Any product that may be evaluated in this article, or claim that may be made by its manufacturer, is not guaranteed or endorsed by the publisher.
